# Deciphering the Molecular Mechanism of Yifei-Sanjie Pill in Cancer-Related Fatigue

**DOI:** 10.1155/2023/5486017

**Published:** 2023-02-13

**Authors:** Yingchao Wu, Shuyao Zhou, Dajin Pi, Yangyang Dong, Wuhong Wang, Huan Ye, Zhongjia Yi, Yiliu Chen, Lizhu Lin, Mingzi Ouyang

**Affiliations:** ^1^School of Traditional Chinese Medicine, Jinan University, Guangzhou, Guangdong 510632, China; ^2^College of Forestry and Landscape Architecture, South China Agricultural University, Guangzhou, Guangdong 510642, China; ^3^Guangdong Metabolic Diseases Research Center of Integrated Chinese and Western Medicine, Guangdong Pharmaceutical University, Guangzhou, Guangdong 510006, China; ^4^Oncology Center, The First Affiliated Hospital of Guangzhou University of Chinese Medicine, Guangzhou, Guangdong 510405, China

## Abstract

**Background:**

The incidence of cancer-related fatigue (CRF) is increasing, but its lack of clear pathogenesis makes its prevention and treatment difficult. Therefore, it is of great significance to clarify the pathogenesis of CRF and find effective methods to treat it.

**Methods:**

The CRF model was established by intraperitoneal injection of LLC cells in ICR mice to explore the pathogenesis of CRF and verify the therapeutic effect of the Yifei-Sanjie pill (YFSJ). The active components of YFSJ were found by LC/MS, the in vitro inflammatory infiltration model of skeletal muscle was constructed by TNF-*α* and C2C12 myoblasts, and the results of in vivo experiments were verified by this model.

**Results:**

Behavioral analysis results showed that YFSJ alleviated CRF; histological examination results showed that YFSJ could reverse the tumor microenvironment leading to skeletal muscle injury; ELISA and RNA-seq results showed that the occurrence of CRF and the therapeutic effect of YFSJ were closely related to the tumor inflammatory microenvironment; IHC and WB results showed that the occurrence of CRF and the therapeutic effect of YFSJ were closely related to the Stat3-related signaling pathway and autophagy.

**Conclusions:**

YFSJ can reduce the level of inflammation in the tumor microenvironment in vivo, inhibit the abnormal activation of the Stat3/HIF-1*α*/BNIP3 signaling pathway induced by tumor-related inflammation, thereby inhibiting the overactivation of mitophagy in skeletal muscle, and finally alleviate CRF. Quercetin, one of the components of YFSJ, plays an important role in inhibiting the phosphorylation activation of Stat3.

## 1. Introduction

With the development of society and the improvement of medical level, people's life expectancy is getting longer and longer [1]. Cancer, as a disease closely related to the aging of the body, is accompanied by an increase in life expectancy, and the incidence of cancer is increasingly high [2, 3]. Although the lesions of cancer are mostly confined to one or more places, in the process of their occurrence and development, they have an obvious influence on the whole body. In the process of cancer development, there are a variety of unpleasant symptoms, such as fatigue, weight loss, and pain. The incidence of cancer-related fatigue (CRF) has been reported to be as high as 82% in cancer patients, and tumor burden contributes to varying degrees of fatigue with or without chemotherapy [4]. Unfortunately, the pathogenesis of CRF is not fully understood, and there is no standard treatment.

Previous studies have found that the mechanism of CRF is closely related to tumor-induced overactivation of mitophagy in skeletal muscle [4]. There are many reasons for excessive mitophagy, such as activation of inflammatory factors, oxidative stress, and energy metabolism disorders [5–7]. Tumor burden will also lead to changes in the body's internal environment, such as changes in the immune microenvironment and energy metabolism level [8, 9]. However, the mechanism by which tumor burden leads to mitophagy and fatigue remains unclear.

The Yifei-Sanjie pill (YFSJ), a traditional Chinese patent medicine, can be used to alleviate CRF in clinical practice [10–12]. The mechanism may be to alleviate CRF by reducing skeletal muscle damage [4, 13]. However, the specific molecular mechanism by which YFSJ attenuates skeletal muscle injury in the tumor microenvironment has not been revealed. Therefore, it is of great significance to clarify the relationship between the occurrence of CRF and mitophagy and to reveal the molecular mechanism of YFSJ in the treatment of CRF. In addition, meta-analysis and network pharmacology predicted that quercetin may play an important role in alleviating CRF, but its specific molecular mechanism has not been revealed [14]. Therefore, we will further study the pathogenesis and treatment of CRF.

In this study, we demonstrated that the tumor inflammatory microenvironment leads to the abnormal activation of the Stat/HIF-1*α*/BNIP3 signaling pathway in skeletal muscle cells, which induces excessive mitophagy in skeletal muscle and ultimately leads to fatigue. Several miRNAs have previously been reported to regulate these pathways, but the mechanisms are unclear [15]. At the same time, we confirmed that YFSJ could reduce tumor-induced elevated levels of inflammation in vivo, thereby inhibiting excessive mitophagy in skeletal muscle and ultimately alleviating CRF. Meanwhile, we also found that quercetin, a major component in YFSJ, can specifically inhibit the phosphorylation activation of Stat3, thereby inhibiting TNF-*α*-induced mitophagy, which provides a new paradigm for the modern development and application of traditional drugs.

## 2. Materials and Methods

### 2.1. Animal Ethics Statement

Female ICR mice weighing between 20 and 22 g and aged 4 weeks (Charles River Laboratories, Zhejiang, China) were used in this investigation. All experiments were conducted according to the relevant laws and institutional guidelines. Mice were individually housed in independent ventilated cages at 24°C to 26°C under constant humidity with a 12 h light/dark cycle. Based on clinical practice, the mice were divided into 4 groups (*n* = 10), namely, control, CRF, low, and high groups, in a random order. Permission for the experimental scheme from the Laboratory Animal Ethics Committee of Jinan University was granted (Approval No. 20220301-15).

### 2.2. YFSJ Preparation

YFSJ (Cat. #Z20190015000) was purchased from the First Affiliated Hospital of Guangzhou University of Chinese Medicine (Guangdong, China). Each packet of YFSJ was 8 g, which was equivalent to 16.4 g of herbal medicine. The dosage of the subsequent experiments was the amount of herbal medicine.

### 2.3. Antibodies

Primary antibodies against Stat3 (Cat. #9139T), Phospho-Stat3 (P-Stat3, Cat. #9145T), HIF-1*α* (Cat. #36169T), Atg7 (Cat. #8558T), LC3A/B (Cat. #12741S), CD68 (Cat. #97778S), CD206 (Cat. #24595T), GAPDH (Cat. #5174S), rabbit (Cat. #7074P2), or mouse (Cat. #7076P2) and secondary antibodies were purchased from Cell Signaling Technology (Danvers, MA, USA). Primary antibodies against BNIP3 (Cat. #68091-1-Ig), Beclin 1 (Cat. #11306-1-AP), and p62 (Cat. #18420-1-AP), and COX IV (Cat. #66110-1-Ig) were purchased from Proteintech (Wuhan, China).

### 2.4. Cell Culture

Lewis lung cancer (LLC) cells were acquired from the Guangzhou University of Chinese Medicine (Guangzhou, China). C2C12 myoblasts were purchased from Fuheng BioLogy (Shanghai, China). Dulbecco's modified Eagle's medium, high glucose, L-glutamine, phenol red (DMEM, Cat. #11965092), fetal bovine serum (FBS, Cat. #10270106), penicillin/streptomycin (Cat. #10378016), trypsin-EDTA, and 0.05% phenol red (Cat. #25200072) were purchased by Gibco (NY, USA). All cells were identified by short tandem repeat (STR). Cells were cultured in DMEM supplemented with 10% FBS, 100 U/mL penicillin, and 100 *μ*g/mL streptomycin. Cells are placed in a three gas incubator (Thermo Fisher Scientific, Waltham, MA, USA) containing 5% CO_2_, 37°C constant temperature, and damp environment for culture. The medium was changed every 72 hours, and the cells were routinely subcultured when they reached 90% confluence. Logarithmic growth phase LLC and C2C12 cells were used to conduct the experiments. In order to maintain the stability of HIF-1*α* protein in vitro, C2C12 cells were supplemented with 0.1 *μ*M DMOG (Cat. #D3695, Sigma-Aldrich, Darmstadt, Germany) in an in vitro experiment.

### 2.5. In Vivo Inflammatory CRF Model

**Figure 1:**
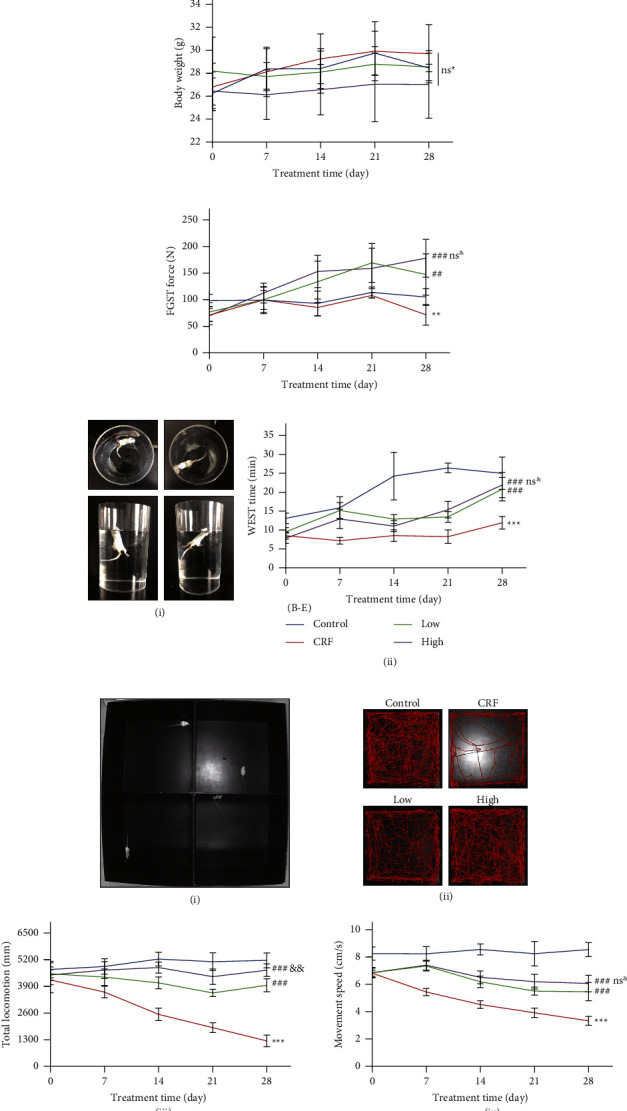
YFSJ attenuates tumor-induced fatigue. (a) The in vivo experimental protocol. (b) Body weight was measured every 7 days. (c) The FGST force. (d) WEST; (i) schematic diagram of WEST; (ii) the WEST time. (e) OFT; (i) schematic diagram of OFT; (ii) the OFT track map; (iii) the OFT movement distance; (iv) the OFT movement speed. The data are presented as means ± SD; ^ns^^*∗*^*p* > 0.05, ^*∗∗*^*p* < 0.01, and ^*∗∗∗*^*p* < 0.001 compared with the control group; ^##^*p* < 0.01 and ^###^*p* < 0.001 compared with the CRF group; ^ns&^*p* > 0.05 and ^&&^*p* < 0.01 compared with the low group; 0, 7, and 14 days, *n* = 10; 21 and 28 day, *n* = 6.

We induced an inflammatory CRF model in mice via intraperitoneal injection of LLC cells following the published protocols [16]. After acclimation for 7 days, mice in groups CRF, Low, and High were intraperitoneally injected with 1 × 10^7^ LLC cells/100 *μ*L each mouse. The experimental protocol is shown in Figure 1(a). Three days after LLC cell inoculation, treatment was initiated, and the dose of YFSJ was referred to in our previous report [4]. The control and CRF groups were given normal saline intragastric administration (0.2 ml/d) for 28 days. Low group was given 2 g/kg/d YFSJ by gavage, 0.2 ml, for 28 days. High group was given 4 g/kg/d YFSJ by gavage, 0.2 ml, for 28 days.

### 2.6. Behavioral Tests

#### 2.6.1. Forelimb Grip Strength Test (FGST)

The muscle strength of mice was measured by FGST according to the published protocol [17]. In short, we first attached a grid to a force transducer, then let the mouse grasp the grid tightly with both forepaws, and then pulled the mouse away from the grid, breaking its grasp. The transducer records the maximum force applied by the mouse on the grid during the pull. Let the mouse pull the grid three times in a row, and take the average of the three pulls.

#### 2.6.2. Weight-Loaded Exhaustive Swimming Test (WEST)

The muscle exercise endurance of mice was measured by WEST according to the published protocol [18]. In short, the mice were individually loaded with a lead (7% of body weight) on their tail root and placed in the swimming pool (diameter 30 cm × height 50 cm) filled with 30 cm deep water (maintained at 25 ± 1°C). The exhaustive swimming time was recorded as described in the previous study [19]. Considering that the mice could not fully recover their physical strength before the test in a short time, the test was not repeated for each mouse in a single test.

#### 2.6.3. Open Field Test (OFT)

The locomotor willingness and ability of mice were measured by OFT according to the published protocol [20]. In short, the OFT was conducted in an arena made of plexiglass (100 × 100 × 50 cm^3^). Each mouse was placed in the center of the apparatus and analyzed for 10 min in a quiet room. The total movement distance and movement speed were calculated and analyzed using the EthoVision XT 14 software (Noldus Information Technology Co., Ltd., Beijing, China). In consideration of the adaptation of mice to the experimental environment, the test was not repeated for each mouse in a single test.

### 2.7. Mouse Euthanasia and Sample Collection

According to the corresponding experimental plan, the mice were anesthetized with pentobarbital (150 mg/kg, i.p.) after all the corresponding behavioral tests were completed (four mice at 14 days and six at 28 days). In order to maintain a sufficient number of mice for behavioral analysis, only 4 mice from each group were sampled at 14 days. The plasma was collected, the mice were euthanized by cervical dislocation, and the lung, liver, spleen, and gastrocnemius muscle tissues were removed for subsequent experiments. The plasma and tissues collected will be pretreated differently depending on the subsequent experiments.

### 2.8. Histological Analysis

#### 2.8.1. Hematoxylin and Eosin (HE) Staining

HE-stained sections of lung, liver, and kidney were prepared according to previously reported methods [21]. The stained slices were observed and photographed under a light microscope at 100× or 40× magnification (NIKON Eclipse ci, Japan). For quantification of immunostaining intensities, Image *J* software (National Institutes of Health, Bethesda, MD, USA) was used, as stated by Sysel et al. [22]. The inverse mean density was determined, as reported by Vis et al. [23], in 1 randomly chosen field from various sections of 3 mice in each group.

#### 2.8.2. Transmission Electron Microscope (TEM)

TEM sections of gastrocnemius muscle were prepared according to previously reported methods [4]. The slices were observed and photographed under an electron microscope (HITACHI HT7700, Japan).

#### 2.8.3. Immunohistochemical (IHC) Staining

IHC-stained sections of gastrocnemius muscle were prepared according to previously reported methods [21]. Primary antibodies include Beclin 1 (1 : 100), LC3 (1 : 500), p62 (1 : 1000), CD68 (1 : 300), and CD206 (1 : 400). Images were captured under a light microscope (Leica, Germany).

### 2.9. Enzyme-Linked Immunosorbent Assay (ELISA)

Mouse IL-6 ELISA kit (Cat. #EK206/3-48) and mouse TNF-*α* ELISA kit (Cat. #EK282/4-48) were purchased from Multisciences (Zhejiang, China). The serum IL-6 and TNF-*α* contents of mice were measured according to the instructions of the kit.

### 2.10. RNA-Seq

The mouse gastrocnemius muscle RNA-seq assay was performed as previously reported [21]. The differential expression analysis and differential gene enrichment analysis (Gene Ontology, GO, and Kyoto Encyclopedia of Genes and Genomes, KEGG), and visualized, were performed on the Novo Magic (URL: https://magic.novogene.com) of Beijing Novogene Biotechnology Co., LTD.; *n* = 3. Datasets for RNA-seq can be obtained from the Sequence Read Archive at the NCBI (URL: https://www.ncbi.nlm.nih.gov/sra/PRJNA874361).

### 2.11. Western Blotting (WB)

WB analysis of gastrocnemius muscles was performed as previously reported [21]. If mitochondrial protein isolation is required, it is done using the mitochondrial protein extraction kit (BB-3171, Best Bio, Shanghai, China). Primary antibodies include Stat3 (1 : 1000), P-Stat3 (1 : 1000), HIF-1*α* (1 : 1000), BNIP3 (1 : 5000), Beclin 1 (1 : 1000), Atg7 (1 : 1000), LC3A/B (1 : 1000), p62 (1 : 1000), GAPDH (1 : 2500), and COX IV (1 : 2000). After the primary antibody protocol was completed, the corresponding secondary antibody (1 : 5000) was added according to the protocol. The density values of the bands were captured and documented through a gel image analysis system (ChemiDox™, Bio-Rad, USA) and normalized to GAPDH or COX IV; *n* = 3.

### 2.12. Q-Orbitrap High Resolution Liquid/Mass Spectrometry (LC/MS)

As previously reported [4], we identified the components contained in YFSJ by LC/MS. This part was completed by Wuhan Servicebio Company (Hubei, China).

### 2.13. Access to Active Ingredients and Molecular Docking

LC/MS results were used in conjunction with previously reported methods [24] to obtain the active components in YFSJ. Using the TCM Network Pharmacology Analysis System (TCMNPAS, URL: https://54.223.75.62:3838/), quercetin was used as ligands and Stat3 as receptors for molecular docking, and their binding sites and binding affinity were analyzed. Protein docking pocket parameters were obtained from ligands, and the lower the value of “affinity” (the greater the absolute value), the stronger the binding force. Finally, all the docking results were analyzed by “RMSD,” and an RMSD value less than 2 was considered reliable.

### 2.14. MTT Colorimetric Assay

Cell viability was measured by the MTT colorimetric assay according to the published protocol [21]. Thiazolyl blue tetrazolium bromide (MTT, Cat. #V13154) was supplied by Gibco (NY, USA). If the experimental process needs to be treated with the autophagy inhibitor 3-MA (Cat. #M9281, Sigma-Aldrich, Darmstadt, Germany), the use method is pretreatment for 4 hours. All other factors were treated for 48 hours. IL-6, Mouse (Cat. #CZ52157-EA) and TNF-*α*, Mouse (Cat. #CZ52347-EA) were purchased from Shanghai Yingxin Laboratory Equipment Co., Ltd. (Shanghai, China). Quercetin (Cat. #S25567) was purchased from Shanghai Yuanye Bio-Technology Co., Ltd. (Shanghai, China). *n* = 6.

### 2.15. MDC Staining Assay

Cell autophagy was measured by the MDC staining assay according to the manufacturer's instructions. Autophagy staining assay kit with MDC (Cat. #C3018S) was supplied by Beyotime Biotechnology (Shanghai, China). The fluorescence intensities were measured at an emission wavelength of 512 nm and an excitation wavelength of 335 nm using a fluorescence microplate reader (BioTek, Vermont, USA). The data are expressed as the percentage of the fluorescence intensity relative to that of the control group, *n* = 6.

### 2.16. Statistical Analysis

The experimental data were analyzed using Student–Newman–Keuls (*S*-*N*-*K*) in ANOVA with SPSS version 13.0 software (SPSS Inc., IL, USA) and GraphPad Prism 9 (GraphPad Software, LLC, California, USA) to graph the data. The results are presented as the mean values ± standard deviation (SD). A *p* value <0.05 was considered statistically significant.

## 3. Results

### 3.1. YFSJ Improves the Exercise Ability of CRF Mice

The in vivo experiment protocol is shown in Figure 1(a). In order to explore the dynamic changes of biochemical indexes in vivo during tumor development, mice were sampled at two time points in this experiment. At the end of the experiment, there was no significant difference in body weight between the groups (Figure 1(b)). The results of FGST, reflecting exercise intensity; WEST, reflecting exercise endurance; and OFT, reflecting exercise willingness and ability, showed that all aspects of the exercise level indexes in the CRF group were significantly lower than those in the control group (Figures 1(c)–1(e)). At the beginning or after the beginning of the treatment, each fatigue index of mice gradually showed the fatigue state of mice. After the YFSJ treatment, all the indexes reflecting exercise were significantly improved. However, there was no significant difference in the effect of low-dose YFSJ and high-dose YFSJ, and only the total locomotion distance of the high group was higher than that of the low group in the OFT. These behavioral exercise indicators are important indicators to evaluate fatigue in various aspects. Therefore, our data show that the CRF model is successfully constructed, and YFSJ can effectively alleviate CRF.

### 3.2. YFSJ Reversed Cancer-Induced Mitochondrial Damage in Skeletal Muscle and Inflammation Levels

**Figure 2:**
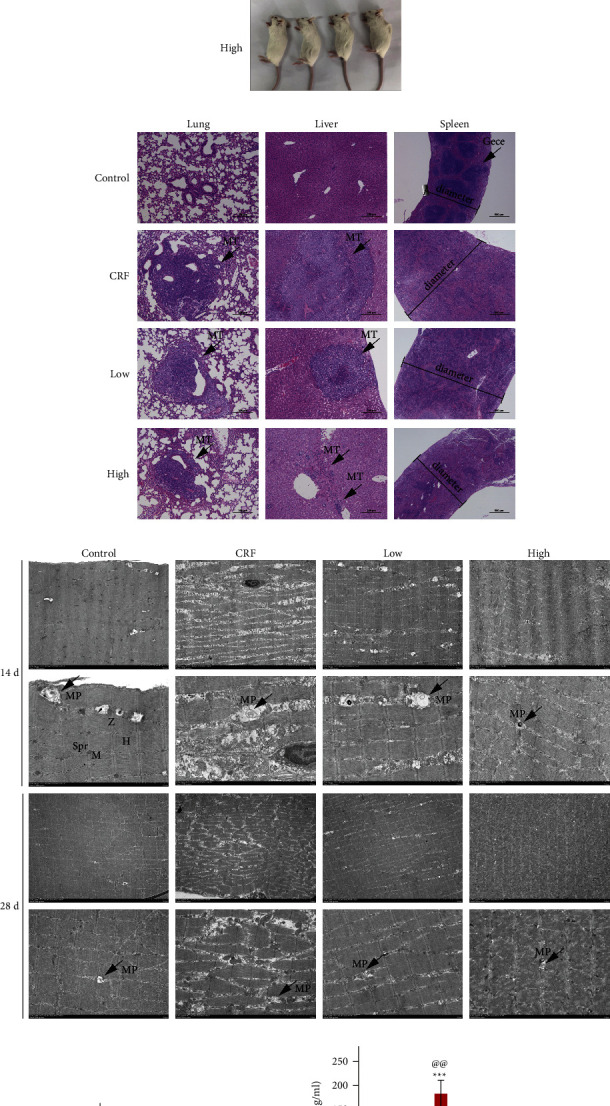
YFSJ reversed cancer-induced mitochondrial damage in skeletal muscle and inflammation levels. (a) Pictures of 28 day mice before sample collection. (b) Representative HE staining slice images of lung, liver, and spleen from 28 day mice; lung and liver, scale bars = 200 *μ*m; spleen, scale bars = 500 *μ*m; MT means metastasis; gece means germinal center. (c) Representative TEM slices images of skeletal muscle; scale bars = 5 *μ*m or 2 *μ*m; MP means mitophagosome; Spr means sarcoplasmic reticulum; *M* means mitochondrion; *Z* means *Z* disk; *H* means *H* band. (d) and (e) IL-6 and TNF-*α* concentrations in mice serum; data were presented as mean ± SD; ^*∗∗*^*p* < 0.01 and ^*∗∗∗*^*p* < 0.001 compared with the control group; ^#^*p* < 0.05, ^##^*p* < 0.01, ^###^*p* < 0.001 compared with the CRF group; ^&^*p* < 0.05 and ^&&^*p* < 0.01 compared with the low group; ^ns@^*p* > 0.05 and ^@@^*p* < 0.01 compared with the 14 day group; *n* = 4.

In order to explore the causes of CRF and the therapeutic effects of YFSJ, we analyzed the samples taken from mice, and the photos of mice before sampling are shown in Figure 2(a). HE staining showed tumor lesions in the lungs and liver of mice after intraperitoneal injection of LLC cells. In addition, LLC cell injection resulted in spleen enlargement and structural disorders (such as germinal center structural changes). High-dose YFSJ treatment could reverse spleen enlargement, but YFSJ has no obvious effect on the reversal of spleen substructural changes (Figure 2(b)). TEM showed that the structure of skeletal muscle fibers was disordered, the sarcoplasmic reticulum was dilated, and the number of mitophagosomes increased significantly in the CRF group. In the CRF group, muscle fiber arrangement was more disordered and structure was more loose at 28 days compared with 14 days. After YFSJ treatment, the number of mitophagosomes in skeletal muscle was significantly reduced, and the morphology of muscle fibers was significantly improved (Figure 2(c)). ELISA results showed that the serum levels of proinflammatory factors, such as IL-6 and TNF-*α*, were significantly increased in the CRF group, and the level of TNF-*α* in the CRF group was significantly higher at 28 days than at 14 days. YFSJ could significantly reverse the increase in proinflammatory factor level caused by the tumor, and the reverse effect was more obvious in the high group than in the low group (Figures 2(d) and 2(e)). Based on these results, we suggested that the occurrence of CRF and the therapeutic effect of YFSJ are highly correlated with the tumor inflammatory microenvironment and skeletal muscle mitophagy.

### 3.3. The Cause of CRF and the Mechanism by Which YFSJ Alleviates CRF Are Closely Related to Inflammation, Mitophagy, and Stat/HIF-1 Signaling Pathway

**Figure 3:**
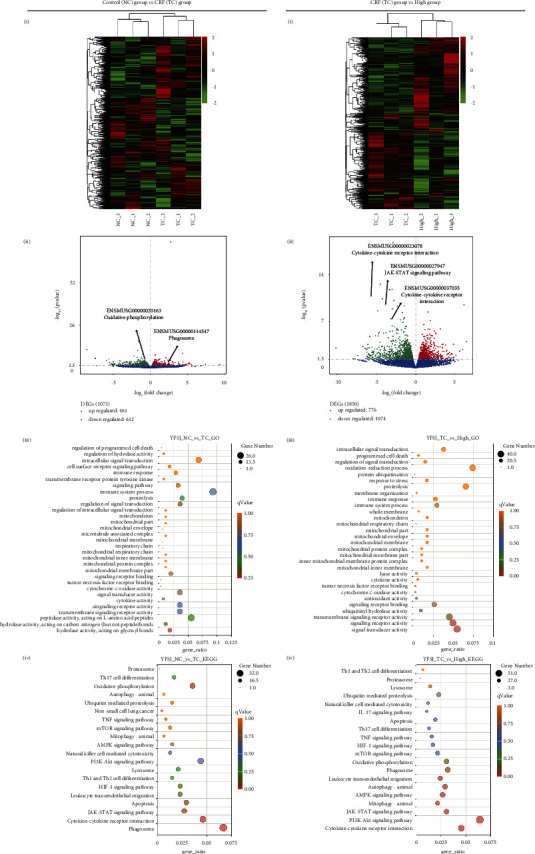
YFSJ treatment on gene expression profiling of skeletal muscle tissues in CRF mice. RNA-seq was performed to determine the DETs in skeletal muscle tissues from each group. (a) Comparison of control group with CRF group. (b) Comparison of CRF group with high group. (i) Hierarchical clustering plots and (ii) volcano plots were used to compare gene expression profiles (|fold change| ≥ 2, *p* < 0.05). (iii) GO enrichment analysis was performed based on the DETs from both comparisons, from top to bottom are BP, CC, and MF, respectively. (iv) KEGG enrichment analysis was performed to identify the enriched signaling pathway in both comparisons *n* = 3.

To explore the potential mechanism of YFSJ in alleviating CRF, RNA-seq was performed to identify the differentially expressed transcripts (DETs) in skeletal muscle tissues among different groups. As shown in Figures 3(a) (i) and 3(a) (ii) (left panels), the CRF group significantly upregulated the expression of 461 transcripts and downregulated the expression of 612 transcripts in skeletal muscle tissues of mice, compared to the control group, while YFSJ treatment significantly upregulated the expression of 776 transcripts and downregulated the expression of 1074 transcripts, compared with the CRF group (Figures 3(b) (i) and 3(b) (ii), right panel).

GO enrichment analysis based on the DETs between control, CRF, and high groups revealed multiple predicted potential functions. As shown in Figure 3(a) (iii), biological process (BP) analysis showed that DETs were mainly associated with intracellular signal transduction, programmed cell death, and cellular immunity. Cellular component (CC) analysis indicated that DETs were mainly involved in the structure of mitochondria. In the molecular function (MF) category, DETs were significantly enriched in signaling receptor binding, cytokine and hydrolase activity, and so on. As shown in Figure 3(b) (iii), BP analysis showed that DETs were associated with intracellular signal transduction, programmed cell death, proteolysis, and immune response. As to CC, DETs were significantly enriched in the mitochondrial membrane part. The MF analysis for these DETs includes lyase and cytokine activity, antioxidant activity, and signal transducer activity.

KEGG pathway analysis based on the DETs between the control and CRF groups revealed multiple enriched signaling pathways, including phagosome, mitophagy, oxidative phosphorylation, TNF signaling pathway, JAK-STAT signaling pathway, and HIF-1 signaling pathway (Figure 3(a) (iv)). Moreover, KEGG pathway analysis based on the DETs between the CRF and YFSJ groups demonstrated multiple enriched signaling pathways, including lysosome, mitophagy, oxidative phosphorylation, TNF signaling pathway, cytokine-cytokine receptor interaction, JAK-STAT signaling pathway, and HIF-1 signaling pathway (Figure 3(b) (iv)). The integrative analysis between the two comparisons (control vs. CRF and CRF vs. high) demonstrated that multiple signaling pathways were enriched in both comparisons. Notably, we find that both cytokine-cytokine receptor interaction, oxidative phosphorylation, and phagosome were significantly enriched in both comparisons, which encouraged us to further explore the regulatory effects of YFSJ on inflammatory cytokines, mitophagy, and activation of the Stat/HIF-1/mitochondria signaling pathway in skeletal muscle tissues of CRF mice.

### 3.4. In Vivo, YFSJ Alleviates CRF by Inhibiting Mitophagy Induced by the Stat3/HIF-1*α*/BNIP3 Signaling Pathway Overactivation in Skeletal Muscle

**Figure 4:**
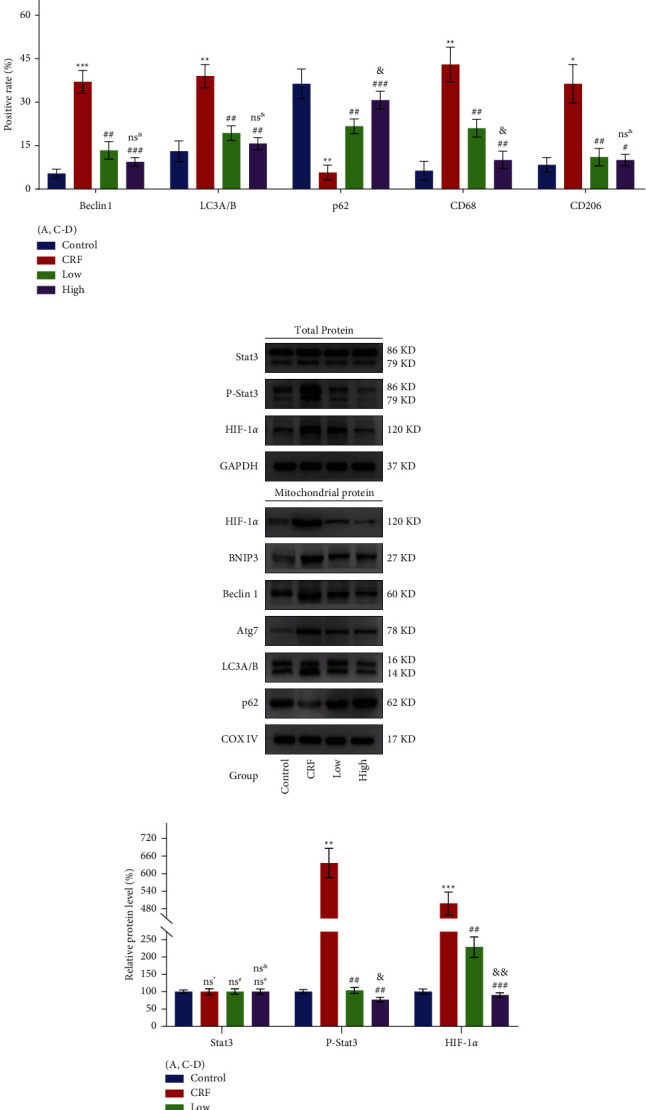
YFSJ alleviates CRF by inhibiting mitophagy induced by the Stat3/HIF-1*α*/BNIP3 signaling pathway overactivation in skeletal muscle. (a) The IHC staining and their relative expression levels were performed to observe proteins associated with autophagy and inflammation in skeletal muscle tissues of mice from each group. (b) WB analysis was performed to determine the protein expression of the Stat3/HIF-1*α*/BNIP3 signaling pathway and mitophagy-related proteins and (c, d) their relative expression levels. GAPDH or COX IV was used as the internal control. Data were presented as mean ± SD; ^ns^^*∗*^*p* > 0.05, ^*∗*^*p* < 0.05, ^*∗∗*^<0.01, and ^*∗∗∗*^*p* < 0.001 compared to the control group; ^ns#^*p* > 0.05, ^#^*p* < 0.05, ^##^*p* < 0.01, and ^###^*p* < 0.001 compared to the CRF group; ^ns&^*p* > 0.05, ^&^*p* < 0.05, and ^&&^*p* < 0.01 compared to the low group; *n* = 3.

Further verification of YFSJ treatment on autophagy and inflammatory infiltration of skeletal muscle tissues by performing IHC staining revealed an increase in the percentage of positively stained Beclin 1, LC3, CD68, and CD206 cells and a decrease in the percentage of positively stained p62 cells in the skeletal muscle tissues of CRF mice, while YFSJ treatment significantly reversed the percentage of positively stained cells (Figure 4(a)). To further confirm the increased level of autophagy in skeletal muscle mitochondria in the CRF group and the effective reversal of excessive mitophagy by YFSJ, total protein and mitochondrial protein were extracted from skeletal muscle tissues for analysis. WB analysis of total protein of skeletal muscle tissue indicated that P-Stat3 and HIF-1*α* protein expression levels were increased in the CRF group, and YFSJ treatment significantly reversed these changes, and the reversal effect in the high group was more obvious than that in the low group. Moreover, WB analysis of mitochondrial proteins in skeletal muscle tissue indicated that HIF-1*α*, BNIP3, Beclin 1, Atg7, and LC3B protein expression levels were increased, while p62 protein expression levels were decreased in the CRF group. Similarly, YFSJ treatment reversed these changes in the CRF group, and the effect was more pronounced in the high group than in the low group (except for p62) (Figures 4(b)–4(d)). Therefore, these results indicated that YFSJ can attenuate tumor-induced inflammatory infiltration and overactivation of mitophagy in skeletal muscle tissue.

### 3.5. Quercetin Is an Active Component of YFSJ Acting on the Stat3-Related Signaling Pathway

**Figure 5:**
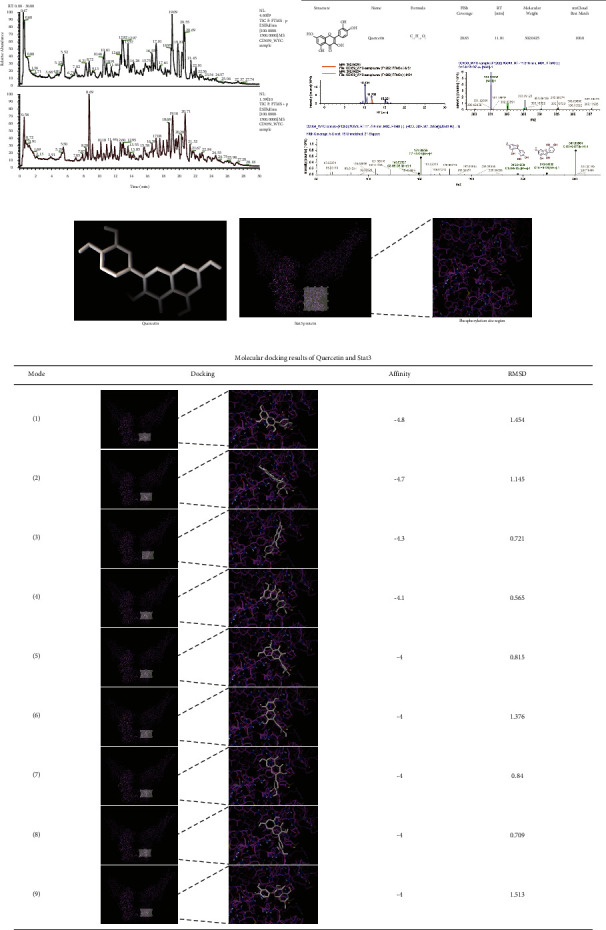
Quercetin is an active component of YFSJ acting on the Stat3-related signaling pathway. (a) Total ion current profiles for identification of natural products in samples (black is the total ion current diagram in negative ion mode and red is the total ion current diagram in positive ion mode). (b) The information of the compound (quercetin) obtained by matching the LC/MS map with the database. (c) The 3D structure of quercetin. (d) The 3D structure and phosphorylation site of Stat3 protein. (e) Molecular docking mode prediction of quercetin and Stat3 and its “affinity” and “RMSD.”

The LC/MS results of YFSJ are shown in Figure 5(a). By comparing the LC/MS results with the database, we obtained quercetin, one of the active components of YFSJ (Figure 5(b)). We used TCMNPAS to perform molecular docking between quercetin and Stat3 protein and verified the docking results, which were visualized using software PMV-1.5.7 (DeLano Scientific LLC). The 3D structures of quercetin and Stat3 protein and its phosphorylation sites are shown in Figures 5(c) and 5(d). Molecular docking results show that the binding site of quercetin to Stat3 is highly coincident with the phosphorylation site of Stat3, and the “absolute value of affinity” is relatively large, indicating that the binding of the two is relatively stable. We conducted RMSD verification on the predicted molecular docking modes of quercetin and Stat3, and the results showed that the RMSD values of all the predicted docking modes are less than 2. This suggests that the binding of quercetin to the phosphorylation site of Stat3 is feasible at the level of molecular interaction. In summary, we predict that quercetin, an active component in YFSJ, can effectively and specifically inhibit Stat3 phosphorylation, thereby inhibiting the overactivation of the Stat3/HIF-1*α*/BNIP3 signaling pathway caused by abnormal phosphorylation of Stat3, and ultimately inhibiting excessive mitophagy.

### 3.6. Quercetin Can Reverse TNF-*α*-Induced Mitophagy in C2C12 Myoblasts

**Figure 6:**
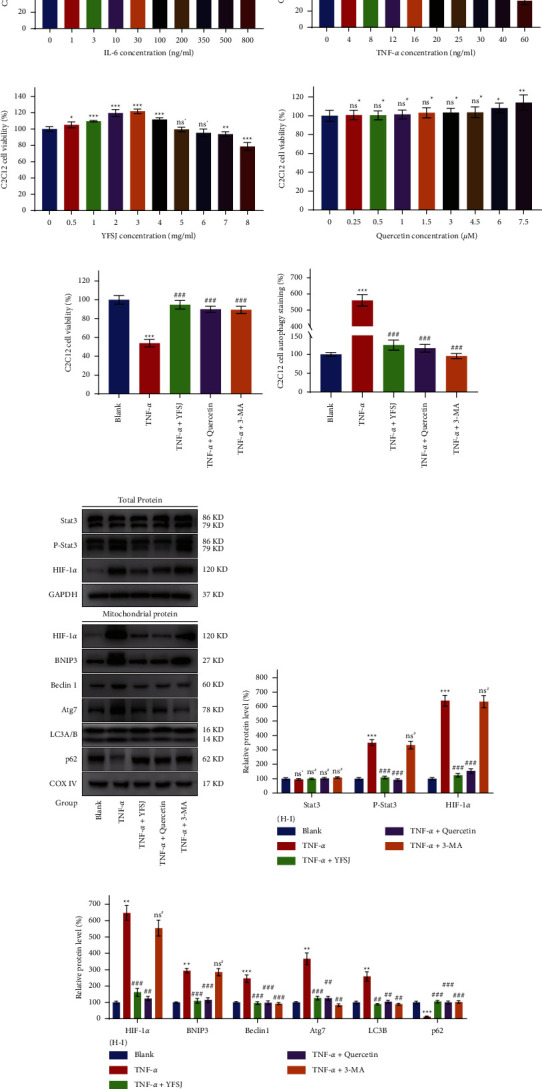
Quercetin can reverse TNF-*α*-induced mitophagy in C2C12 myoblasts. (a–d) Effect of IL-6, TNF-*α*, YFSJ, and quercetin on the viability of C2C12 myoblasts, respectively, *n* = 3. (e) Reverse effect of YFSJ (2 mg/ml), quercetin (1.5 *μ*M), and 3-MA (pretreatment with 2 mM for 4 h) on TNF-*α* (20 ng/ml)-induced decline viability and (f) increase autophagy in C2C12 myoblast, *n* = 3. (g) WB analysis was performed to determine the protein expression of the Stat3/HIF-1*α*/BNIP3 signaling pathway and mitophagy related proteins, and (h, i) their relative expression levels. GAPDH or COX IV was used as the internal control. Data were presented as mean ± SD; ^ns^^*∗*^*p* > 0.05, ^*∗∗*^*p* < 0.01, and ^*∗∗∗*^*p* < 0.001 compared with the blank group; ^ns#^*p* > 0.05, ^##^*p* < 0.01, and ^###^*p* < 0.001 compared with the TNF-*α* group; *n* = 3.

In order to determine which inflammatory factors activate mitophagy and whether quercetin can inhibit mitophagic cell death in skeletal muscle induced by inflammatory factors, we used C2C12 myoblasts to establish an in vitro skeletal muscle model. MTT colorimetric results showed that TNF-*α* inhibited the viability of C2C12 myoblasts much more than IL-6 (Figures 6(a) and 6(b)). Therefore, TNF-*α* was selected for subsequent in vitro experiments. Although both YFSJ and quercetin alone had effects on the viability of C2C12 myoblasts, their effects were minor (Figures 6(c) and 6(d)). Our results showed that YFSJ, quercetin, and 3-MA could reverse the inhibitory effect of TNF-*α* on C2C12 myoblasts to a large extent (Figure 6(e)). 3-MA was used here as a positive control for the inhibition of autophagy. MDC autophagy staining showed that TNF-*α* could significantly induce autophagy in C2C12 myoblasts, which could be reversed by YFSJ, quercetin, and 3-MA (Figure 6(f)). The results of MDC autophagy staining would be even more significant if considering the decrease in cell number caused by TNF-*α* inhibition of C2C12 myoblast proliferation.

To further confirm that TNF-*α* can induce mitophagy in C2C12 myoblasts, thereby inhibiting the proliferation of C2C12 myoblasts. We used WB to analyze the total protein and mitochondrial protein of C2C12 myoblasts treated with each factor. WB results showed that the expressions of P-Stat3 and HIF-1*α* in total proteins increased significantly after TNF-*α* treatment, which was reversed by YFSJ and quercetin treatment, but not by 3-MA treatment. Moreover, TNF-*α* treatment increased the protein expression of HIF-1*α*, BNIP3, Beclin1, Atg7, and LC3B and decreased the protein content of p62 in mitochondria. YFSJ and quercetin treatment significantly reversed these changes, and 3-MA also reversed these changes (except HIF-1*α* and BNIP3) (Figures 6(g)–6(i)). These evidence suggest that TNF-*α* can effectively induce mitophagy in C2C12 myoblasts, thereby inhibiting the proliferation of C2C12 myoblasts, while YFSJ and quercetin can restore the viability of C2C12 myoblasts by reversing TNF-*α* induced mitophagy.

## 4. Discussion

CRF is an uncomfortable symptom associated with the occurrence and development of cancer, which seriously affects the quality of life of cancer patients [25]. Because the mechanism of CRF is not completely clear, it brings difficulties in the clinical prevention and treatment of the disease. So far, there is no accepted treatment method for CRF. Ethnomedicine, represented by traditional Chinese medicine (TCM), is considered a potential treatment for CRF because TCM has many prescriptions for fatigue [26, 27]. According to our study, we demonstrated that Chinese medicine YFSJ can effectively alleviate CRF. Meanwhile, the pathogenesis of CRF, the effect target, and the underlying molecular mechanism of YFSJ were revealed.

In the process of tumor growth, the body will produce a lot of proinflammatory factors [28], making the body of cancer patients always in a state of high inflammation level [29]. This result was consistent with our studies. Our in vivo results demonstrated that the growth of tumor cells leads to an increase in the level of proinflammatory cytokines in serum, which in turn increases the level of inflammatory infiltration of muscle cells. YFSJ treatment can effectively reduce the level of proinflammatory factors in serum, thereby reducing the degree of inflammatory infiltration of muscle cells. At the same time, the size of the spleen can also reflect the level of inflammation in vivo to a certain extent [30]. We also found that the spleen of mice was significantly enlarged after tumor cell inoculation and decreased after high-dose YFSJ treatment compared with the CRF group.

Mitochondria are important places for energy production in the body. The low mitochondrial function will lead to a lack of enough energy in the body, resulting in decreased exercise ability and a sense of fatigue [31]. We observed that the number of mitophagosomes was positively correlated with the degree of fatigue in mice. Our data confirmed that after the injection of tumor cells, all aspects of the motor indexes of mice showed varying degrees of decline, which directly indicated that the growth of tumor cells would cause fatigue in the host body. Fortunately, after YFSJ treatment, all exercise indexes reflecting fatigue degree were improved to a great extent, which indicated that YFSJ could effectively alleviate CRF.

High levels of inflammation in the body lead to mitophagy in skeletal muscle [32]. Our previous studies also confirmed that the occurrence of CRF is closely related to the damage of skeletal muscle [4]. Studies have shown that the tumor inflammatory microenvironment can continuously activate STAT3-related signaling pathways [33]. Therefore, we linked inflammation, Stat3 molecular-related signaling pathways, and mitophagy with the occurrence of CRF and the therapeutic effect of YFSJ. Activation by Stat3 phosphorylation promotes HIF-1*α* expression in the cytoplasm [34], and the HIF-1*α* in the cytoplasm binds to the mitochondrial membrane [35], further promoting the expression of BNIP3 in the mitochondria [36]. The BH3 domain of BNIP3 competes with Beclin 1 to bind Bcl-2 or Bcl-xL, resulting in the formation of complexes between Beclin 1 and Class III PI3K, which activates mitophagy. At the mature stage of the autophagosome, BNIP3 can recruit the autophagy-related protein LC3 to the mitochondria. It promotes the formation of autophagosomes and actively participates in the clearance of damaged mitochondria [37]. Atg7 is a key component involved in autophagosome formation [38], and p62 is a marker of autophagic lysosomes. When autophagy occurs, lysosomes are degraded, and the expression of p62 will decrease [39]. Our data confirm that the tumor inflammatory microenvironment in vivo abnormally activates the Stat3/HIF-1*α*/BNIP3 signaling pathway in skeletal muscle, leading to excessive mitophagy, which disrupts the body's energy balance and ultimately leads to fatigue.

To further validate the in vivo results and simplify the experimental model, we treated C2C12 myoblasts with TNF-*α* to mimic the inflammatory infiltration of skeletal muscle in vitro. Consistent with our in vivo results, TNF-*α* significantly induced mitophagy and inhibited cell viability in C2C12 myoblasts. YFSJ and quercetin, the active component of YFSJ, significantly reversed TNF-*α*-induced mitophagy and cell viability inhibition in C2C12 myoblasts. 3-MA is an inhibitor of PI3K. It is widely used as an inhibitor of autophagy by inhibiting class III PI3K [40]. Therefore, it is able to prevent BNIP3 from further activating Beclin1, ultimately inhibiting the occurrence of autophagy. Data showed that 3-MA reversed TNF-*α*-induced autophagy in C2C12 myoblasts. These evidence further confirmed that TNF-*α* plays an important role in activating mitophagy, leading to CRF, and quercetin plays an important role in YFSJ treatment of CRF.

**Figure 7:**
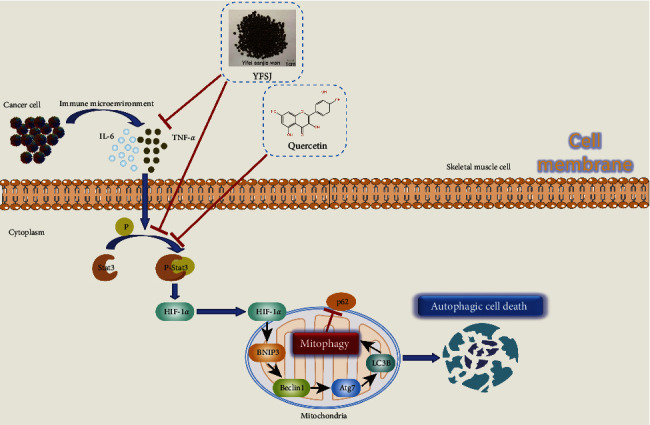
Schematic illustration of the potential underlying mechanism responsible for YFSJ and its component quercetin alleviating CRF.

In summary, YFSJ acts as a traditional Chinese medicine compound, which may alleviate CRF through the action of multiple targets (Figure 7). Firstly, it reduces the level of proinflammatory factors in the body, thereby reducing the degree of inflammatory infiltration of skeletal muscle cells. Secondly, it inhibits the overphosphorylation of Stat3 protein (quercetin, an active ingredient in YFSJ, may be at work here), thereby inhibiting the overactivation of the Stat3/HIF-1*α*/BNIP3 signaling pathway. Finally, it inhibits excessive mitophagy and alleviates CRF under the action of multiple targets. Our results not only confirm that YFSJ is an effective regimen for the treatment of CRF but also reveal the pathogenesis of the disease and, at the same time, provide new clues for the development of drugs with natural compounds from ethnomedicine.

## 5. Conclusions

YFSJ and its component quercetin can inhibit the overactivation of skeletal muscle mitophagy mediated by the abnormal activation of the Stat3/HIF-1*α*/BNIP3 signaling pathway induced by the tumor inflammatory microenvironment, thereby alleviating CRF.

## Data Availability

All data of this study are available from the first author Yingchao Wu and the corresponding author Mingzi Ouyang if needed. Datasets for RNA-seq can be obtained from the Sequence Read Archive at the NCBI (URL: https://www.ncbi.nlm.nih.gov/sra/PRJNA874361).

## Abbreviations

YFSJ: Yifei-Sanjie pill
CRF: Cancer-related fatigue
HE: Hematoxylin and eosin
TEM: Transmission electron microscope
ELISA: Enzyme-linked immunosorbent assay
IHC: Immunohistochemical
WB: Western blotting
LC/MS: High resolution liquid/mass spectrometry.
